# Evaluation of Probiotic Effects on the Growth Performance and Microbiome of Nile Tilapia (*Oreochromis niloticus*) in a High-Density Biofloc System

**DOI:** 10.1155/anu/5868806

**Published:** 2025-01-08

**Authors:** Beatriz P. N. Oliveira, Uthpala Padeniya, Jacob W. Bledsoe, D. Allen Davis, Mark R. Liles, Aya S. Hussain, Daniel E. Wells, Timothy J. Bruce

**Affiliations:** ^1^School of Fisheries, Aquaculture and Aquatic Sciences, Auburn University, Auburn, Alabama 36849, USA; ^2^Department of Animal, Veterinary and Food Sciences, Aquaculture Research Institute, University of Idaho, Moscow, Idaho 83382, USA; ^3^Department of Biological Sciences, Auburn University, Auburn, Alabama 36849, USA; ^4^Department of Forestry and Natural Resources, Purdue University, West Lafayette, Indiana 47907, USA; ^5^Zoology Department, Faculty of Science, Suez University, Suez, Egypt; ^6^Department of Horticulture, Auburn University, Auburn, Alabama 36849, USA

**Keywords:** aquaculture technologies, *Bacillus* spp., microbial communities, probiotics, symbiotic

## Abstract

Biofloc technology is an aquaculture production system that has gained popularity with tilapia production. Probiotics provide benefits for the host and/or aquatic environments by both regulating and modulating microbial communities and their metabolites. When a probiotic feed is combined with a biofloc system, the production amount may be improved through better fish growth, disease resistance, and/or improved water quality by reducing organic matter and stabilizing metrics such as pH and components of the nitrogen cycle. Two research trials measured Nile tilapia (*Oreochromis niloticus*) growth performance and composition of the microbial communities in the water and within the fish fecal material, following feeding with top-coated probiotic treatments. Trial A incorporated tilapia (71.4 ± 4.4 g), and a commercial diet (Control) that was top coated with either *Bacillus velenzensis* AP193 (AP193; 1 × 10^7^ CFU g^1^) and BiOWiSH Feedbuilder Syn3 (BW; 3.6 × 10^4^ CFU g^−1^). In Trial B, juvenile tilapia (5.34 ± 0.42 g) were fed treatment diets top coated with two different concentrations of BiOWiSH Feedbuilder Syn3 at final concentrations of 3.6 × 10^4^ CFU g^−1^ (BWx1) and 7.2 × 10^4^ CFU g^−1^ (BWx2). Tilapia were offered commercial feed (38% protein floating tilapia feed) as a control diet for both trials. Results from both growth trials indicated no differences in growth performance due to the probiotic additions, except for feed conversion ratio (FCR) in Trial B. Both BWx1 and BWx2 showed improved survival, water quality, solids management, and bacterial composition of water and fecal matter. Even though growth performance results presented no significant differences, results could differ based on the probiotic concentration, the route of probiotic administration, or their impact on the microbial community of the biofloc system culture water. Trial results indicated that testing on a larger scale with varied probiotic doses may be necessary to achieve an effective dosage for improving tilapia growth performance.

## 1. Introduction

Aquaculture is one of the fastest growing sectors of animal protein production in the world [1], with tilapia being one of the most popular and highly cultured farmed fish, having an estimated global production of over 5.3 million tonnes in 2022 [2]. These fish are native to Africa and have been spread to different continents mainly for their value as a food crop [3]. Aquaculture technologies have steadily increased the commercial production intensity, resulting in higher levels of inorganic and organic wastes entering the culture system, potentially increasing disease outbreaks and deteriorating the environment [4–6]. Intensive farming conditions expose fish to a diverse spectrum of microorganisms and stressors that increase their vulnerability to infectious diseases [5–7]. Disease outbreaks in aquaculture have caused economic losses of billions of dollars annually and have been cited as a danger to the aquaculture industry's profitability [8].

A possible solution to improve fish production is using biofloc technologies (BFTs), which are considered environment-friendly aquaculture systems [9]. This culture methodology may have advantages in increasing fish production through improved feed utilization and reduced water exchange through the floc-enriched design [10, 11]. BFT consists of a macroaggregation of bacteria, phytoplankton, cyanobacteria, and nutrient detritus, creating a floc suspended in the water column through heavy aeration and mixing [12]. Therefore, the main principle of a biofloc system is the recycling of waste in terms of leftover feed and feces within the system while producing bacterial biomass or “biofloc” particles [13, 14]. One of the advantages of biofloc systems is increased performance and survival rates. This is most likely related to reduced stress caused by improved water quality and nutritional substances contained in biofloc [15], a decrease in pathogen entry [16, 17], and microbial protein acting as a source of protein to fish [14, 18]. The management of BFT systems is based on the carbon (C) and nitrogen (N) ratio, which can be through feed input or different C supplements, such as molasses or sugar cane bagasse [19]. The role of the C:N ratio is to be used as a source of energy (C source) for the heterotrophic bacteria to consume the ammonia in the system [10, 20]. Through the achievement of dense biofloc, the microbial communities may be altered and decrease the abundance of opportunistic pathogens [21]. Thus, previous studies have investigated the use of specific dietary additives on the health of tilapia reared in biofloc systems [22, 23], and this is a promising new method of enhancing fish health for production stocks.

The prolonged and improper use of antibiotics can lead to pathogen resistance [24]. If the use of antibiotics is to be decreased or eliminated, there is a need to find alternative management strategies to minimize and control disease outbreaks. One management strategy is using probiotics [25]. Gibson et al. [26] defined probiotics as “live microorganisms that, when administered in sufficient concentrations, can confer beneficial results.” The source of probiotics can be exogenous or indigenous, recurring naturally in different hosts [27].

Probiotics work through multiple mechanisms in aquatic animal organisms, including immunomodulation or competitive exclusion, that exert their activity through interaction with the gastrointestinal (GI) mucosa [28–30]. According to Standen et al. [31], the GI tract has an imperative role due to being the organ where probiotics will establish and perform their effects. The microbial communities found in the GI are most affected by feed and rearing conditions [32–34]. Microbial additives function via modulating the gut microbiota and reducing pathogenic bacteria [35], potentially resulting in an increase in feed consumption, absorption of nutrients, and enhanced immunity [36–39]. Thus, they can benefit the host by enhancing growth performance, water quality, immune system response, disease response, and nutrient availability for zooplankton [29, 40].

Probiotics are utilized for several reasons dictated by application and species of choice. Probiotics can be applied to an aquaculture system via feed (pellet feed with probiotics or live food with probiotics) or added to the water column. In aquaculture, they are commonly used as feed additives due to most of the probiotics being destined for the GI tract [41]. The *Bacillus* spp. are the most common probiotic and are considered effective for enhancing the growth and health of aquatic species [6, 42] and have been shown to modulate the microbial community within the fish [43]. For instance, Wu, Liu, and Hu [44] experimented with a *Bacillus* spp. probiotic strain administered as a feed additive with different inclusion levels. This experiment showed that *Bacillus safensis* increased growth, expression of growth-related genes, and immune responses in Nile tilapia. Immune enhancements have also been shown in several other finfish species by incorporating *Bacillus* spp. as probiotics [45, 46].

The current study explored the influence of two probiotics on growth performance, water quality and changes to the gut microbiome of Nile tilapia reared under biofloc conditions. The study results will add to our understanding of how to improve sustainable aquacultural approaches and provide a clearer picture of how probiotics influence the bacterial composition and diversity within the fish intestine and biofloc water.

## 2. Materials and Methods

### 2.1. Experimental System

Two trials were conducted in a greenhouse-based decoupled aquaponics system at the E. W. Shell Fisheries Center at Auburn University, Auburn, Alabama, in agreement with the Auburn University animal care policy (PRN 2022-5068). The research design consisted of nine 1000-gal (3.8 m^3^) cylindrical polypropylene tanks connected to two 500 gallons (1.9 m^3^) reservoir tanks, Aquadyne bead filter (0.2 m^2^ media, 0.6 m × 1.1 m; source), and a 0.25-hp circulation pump (Cascade-PerformancePro Pumps, Hillsboro, OR). Each culture tank was equipped with one (120 cm) long diffuser tubing (rubber/polyethylene diffuser hose, Pentair Aquatic Eco-Systems, Inc, Apopka, FL) hooked to a standard regenerative blower (1.5 hp, Sweetwater—Pentair Aquatic Ecosystems, Inc., Apopka, FL). To control for solids accumulation, each culture tank was equipped with a 114 L conical settling chamber that received water via a powerhead (Maxi-Jet 110 gph), which returned settled water to the culture tank. During the acclimation period, the system was used as a common recirculating aquaculture system (RAS) to allow the development and equalization of the biofloc community across all tanks [47].

### 2.2. Growth Trial

#### 2.2.1. Trial A: Evaluation of Different Probiotics

Prior to the start of the experiment, Nile tilapia (150 tank^−1^) were stocked and acclimated into nine, 3785 L tanks in a recirculating system for a period of 26 days. The fish were fed with commercial feed twice daily, at 08:00 and 16:00 h. At the start of the 109-day growth trial, fish were harvested, sorted for uniformity, counted, weighed, and restocked at a density of 120 fish tank^−1^ (mean initial weight 71.43 ± 4.44 g). The dietary treatments were randomly assigned to fish in each 3785 L tank (as above), and the system switched to operate as individual tank biofloc systems the day after stocking. A commercial feed (Control; 38% protein Optimal Aquafeed-Tilapia G3 floating tilapia feed, Optimal Aquafeed, Omaha, NE) was used throughout the acclimation period and the trial. The commercial feed was sprayed separately with two probiotics, *B. velezensis* strain AP193 (AP193) or BiOWiSH Feedbuilder Syn3 (BW; BiOWiSH Technologies Cincinnati, OH, USA—*B. subtilis*) as a topcoat prior to feeding. The three treatments (i.e., Control, AP193, and BW) were randomly assigned to three replicate tanks. Tilapia were offered experimental diets at 4% body weight, and the feeding ration was adjusted every 4 weeks based on fish growth and feeding response. Five fish per tank were collected as an initial sample for proximate analysis, and individual weight and length were recorded at the start of the trial.

#### 2.2.2. Trial B: BiOWiSH Feedbuilder Syn3 Concentrations Evaluation

The system was stocked with fingerling tilapia (200 tank^−1^) for Trial B and acclimated in nine tanks for 5 days. At the start of the growth trial, the fish were counted and weighed (mean initial weight 5.34 ± 0.42 g). Dietary treatments were randomly assigned to fish in each tank, and the system switched to individual biofloc systems after stocking. The trial used two commercial feeds (45% protein-Optimal Starter #3 and 38% protein-Optimal Aquafeed-Tilapia G3; Optimal Aquafeed, Omaha, NE) as a Control group throughout the trial, as the tilapia were smaller than in Trial A and required a different pellet size initially. The commercial feed (i.e., Control group diet) was top coated with BiOWiSH Feedbuilder Syn3 (BiOWiSH Technologies Cincinnati, OH, USA—*B. subtilis*) in two different concentrations (BWx1 at 3.6 × 10^4^ CFU g^−1^ and BWx2 at 7.2 × 10^4^ CFU g^−1^). The three dietary treatment groups (i.e., Control, BWx1, and BWx2) were randomly assigned to triplicate tanks for the feeding trial. As in Trial A, fish were offered experimental diets at 4% body weight, and the feeding ration was adjusted every 2 weeks based on fish growth and feeding response. A total of 10 fish per tank were collected as an initial sample for proximate analysis, and individual weight and length were recorded at the start of the trial.

### 2.3. Preparation of Probiotic Top-Coated Diets

#### 2.3.1. Trial A: Evaluation of Different Probiotics

A 38% protein floating commercial diet (Optimal Aquafeed Tilapia Grower-G3) was used throughout the study for all treatments. The diet was sent to the University of Missouri Agriculture Experiment Station Chemical Laboratories (Columbia, MO) for proximate composition analysis (Table 1). BiOWiSH Feedbuilder Syn3 (*B. subtilis*) is a water-soluble probiotic that was used to topcoat the commercial pelleted feed at a manufacturer-recommended concentration of 200 g ton^−1^ of feed, which yields a final concentration of 3.6 × 10^4^ CFU g^−1^, that were mixed with distilled water (>2 L/T) for application. After the solution preparation, the feed was loaded into a conveying paddle mixer, sprayed with the probiotic solution, and left to air dry until ready to use. For *B. velezensis* AP193, the commercial spore suspension (1.3 × 10^10^ CFU g^−1^) was prepared and top coated at 8% (w/v) following the same top-coating procedure as the BiOWiSH product to obtain a concentration of 1 × 10^7^ CFU g^−1^ of feed. Feed was stored at 4°C until use.

#### 2.3.2. Trial B: BiOWiSH Feedbuilder Syn3 Concentrations Evaluation

In Trial B, two commercial feeds were used. At the beginning of the trial, fish were offered 2 mm–46% protein tilapia feed, Optimal Starter #3—Optimal Aquafeed (Omaha, NE), and in the fourth week, due to fish growth, the feed was increased to 3 mm—38% protein floating feed—Optimal Aquafeed-Tilapia G3, Optimal Aquafeed (Omaha, NE). Two different concentrations of BiOWiSH Feedbuilder Syn3 (*B. subtilis*) were evaluated. The stock contained 7.2 × 10^7^ CFU g^−1^ which was suspended in distilled water, according to the manufacturer's specifications the stock solution was then applied to the feed as previously described for the BWx1 (3.6 × 10^4^ CFU g^−1^) and BWx2 (7.2 × 10^4^ CFU g^−1^) treatments.

### 2.4. Growth Performance Measures and Feed Utilization

During Trial A, a total of 30 fish were sampled every 4 weeks to follow growth and estimate total tank biomass. The daily ration was adjusted based on growth and feeding response. For Trial B, an average of 50 fish tank^−1^ was sampled every 2 weeks for biomass weight to determine the fish growth and adjust feeding. Following 109 days (Trial A) or 90 days (Trial B) of culture, fish were counted and weighed to determine the final weight, weight gain, final biomass, survival, and feed conversion ratio (FCR). Five fish per tank were randomly collected, euthanized, packed in sealed bags, and stored in a freezer (−20°C) to determine proximate whole body and mineral composition. A bacterial pathogen challenge trial with *Streptococcus iniae* was also conducted following both trials to assess the health benefits of the probiotic inclusion on the tilapia immune response, and these results are detailed by Padeniya et al. [47]. The calculations (based on [48]) used to assess growth performance are as follows:

  $$\begin{array}{c} \text{Mean weight (g)}=\text{Total weight (g) of fish}/\text{number of fish in the same tank}, \end{array}$$

  $$\begin{array}{c} \text{Weight gain (g)}=\text{W2 - W1}, \end{array}$$

  $$\begin{array}{c} \text{W1}=\text{Initial mean weight and W2}=\text{Final mean weight}, \end{array}$$

  $$\begin{array}{c} \text{Percent weight gain}\left(\text{WG}\%\right)=\left(\text{W2-W1/W1}\times \right)100, \end{array}$$

  $$\begin{array}{c} \text{Survival }\left(\%\right)=\left(\text{Final fish number}/\text{initial fish number}\right)\times 100, \end{array}$$

  $$\begin{array}{c} \text{Feed conversion ratio (FCR)}=\left(\text{Total feed input (g)}/\text{biomass gain (g)}\right)/100, \end{array}$$

  $$\begin{array}{c} \text{Apparent net protein retention}\left(\text{ANPR}\%\right)=\left(\text{Final weight}\times \text{final protein content}\right)-\left(\text{initial weight}\times \text{initial protein content}\right)\times 100/\text{protein intake}. \end{array}$$

### 2.5. Water Analysis, Solids Management, and Sample Collection

Throughout both experiments, dissolved oxygen, salinity, and water temperature were measured twice daily using a YSI-55 digital oxygen/temperature meter (YSI corporation, Yellow Springs, Ohio, USA) and were maintained within an acceptable range for fish culture. Total ammonia nitrogen (TAN) and nitrite-N were measured twice weekly with a YSI 9300 photometer (YSI corporation, Yellow Springs, Ohio, USA). During the study, the system pH was monitored twice weekly with the pHTestr30 (Oakton Instrument, Vernon Hills, IL), and an amount of sodium bicarbonate (NaHCO_3_) was added to raise the pH, as needed [49]. To quantify solids production (effluent) in both trials, each tank was equipped with an independent conical bottom settling chamber. Once a month, settleable solids were discharged, collected into a bucket, and the slurry quantified, using a methodology based on Gaona et al. [50]. A homogenized subsample was taken and placed in a large crucible and dried in the oven at 105°C overnight. Dry matter was recorded for each of the nine crucibles. To determine ash, 1 g of sample was placed in a small crucible and combusted in a muffle furnace (ThermoFisher Scientific, Asheville, NC) at 600°C for 9 h. The samples were cooled, and ash was quantified. The effluent/solids were quantified as follows:

  $$\begin{array}{c} \text{Solids (kg) per liter}=\text{Amount of dry solids}/\text{liter}, \end{array}$$

  $$\begin{array}{c} \text{Solids (kg) per feed input}=\text{ Total amount of solids/total feed input}, \end{array}$$

  $$\begin{array}{c} \text{Solids (kg) per biomass produced}=\text{ Total amount of solids/total biomass}. \end{array}$$

### 2.6. Microbial Composition for Fecal Matter and Water Samples

Samples for gut composition were taken on day 0 and the last day of the trial (Trial A: day 109; Trial B: day 90). On day 0, five fish, and at the end of the trial, five fish from each tank were randomly selected. These fish were euthanized using an overdose of 250 mg L^−1^ of tricaine methane sulfonate (MS-222) buffered to a pH of 7.0–7.5 in culture water. Fish were aseptically dissected, and the distal intestine close to the anus was separated from the rest of the gut and used for analysis. The gut was gently squeezed to ensure any remaining digesta were removed. The collected digesta were transferred to cryotubes and submerged in liquid N before being stored at −80°C until further analysis of gut microbiome communities. Water samples were also collected on day 0 and at the end of the trial. The water samples were filtered through a 0.45 µm bottle top filter (ThermoScientific Nalgene Rapid-Flow, ThermoFisher Scientific, Asheville, NC) using a vacuum filter (Model no. 25228-01 WELCH, Monroe, Louisiana, USA). All the samples were stored at −80°C. Samples were sent for DNA extraction and sequencing and were processed and analyzed with the ZymoBIOMICS Targeted Sequencing Service (Zymo Research, Irvine, CA).

### 2.7. Statistical Analysis

Data were analyzed using SAS (V9.4, SAS Institute, Cary, NC, USA). Growth performance, solids management (dry matter and ash), proximate whole body, and mineral composition of fish were subjected to a one-way ANOVA followed by Tukey's multiple comparison tests to evaluate significant differences among treatment means (*p* < 0.05). Water quality parameters were analyzed using one-way ANOVA followed by a time series analysis. The alpha diversity values (observed and Shannon) of water and fecal samples between different treatments were analyzed using one-way ANOVA and pairwise *t*-tests. The bacterial data were analyzed using R version 4.2.1 (R Foundation for Statistical Computing, Vienna, Austria). Microbiota beta diversity of fecal matter and water samples was assessed at the ASV level using unweighted UniFrac and weighted UniFrac metrics. Bray–Curtis dissimilarity analysis was also incorporated to quantify compositional differences, based on microbe counts, between groups [51]. In addition, beta diversity was also evaluated at the genus level by calculating Bray–Curtis dissimilarity matrices after agglomerating ASVs at the genus level. The vegan package [52] was used to evaluate the homogeneity of dispersion (betadisper). Permutational multivariate analysis of variance (PERMANOVA; BiodiversityR) was conducted on all distance metrics to test for shifts in beta diversity centroids between experimental dietary groups [53]. PERMANOVA on all matrices was done considering the extraction technique as a fixed effect and using type III sum of squares and unrestricted permutation of data with 999 permutations [54]. To compare the abundance of the probiotic bacteria, the abundance of microbes belonging to the genus *Bacillus* was compared between treatments using one-way ANOVA and pairwise *t*-tests. All sequencing reads from these trials are available via the NCBI Sequence Read Archive (SRA) under BioProject # PRJNA1174866.

## 3. Results

### 3.1. Water Quality

#### 3.1.1. Trial A

Water quality parameters for Trial A are presented as mean ± SD among treatments as follows: morning and evening dissolved oxygen (7.37 ± 0.64 mg L^−1^, 7.21 ± 1.42 mg L^−1^), temperature (26.84 ± 1.92°C), salinity (0.94 ± 0.44 ppt), pH (6.83 ± 0.66), total ammonia N (6.58 ± 8.48 mg L^−1^), and nitrite (0.6 ± 0.5 mg L^−1^; Table 2). Evaluation of the data, pooled across time, pooled by week, or as time series analysis, showed no difference (*p* > 0.05) between treatments for dissolved oxygen (mg L^−1^), salinity (ppt), pH, and nitrite (mg L^−1^). However, in the case of temperature (°C), there was a significant difference in the BW-fed tilapia (26.90 ± 2.14 °^;^C) compared to the Control (26.64 ± 1.82°C) and AP193 (26.56 ± 1.76°C; *p* = 0.040) groups. This was due to one of the tanks situated close to the exit door of the greenhouse. For total ammonia N (mg L^−1^) and unionized ammonia (mg L^−1^), ANOVA detected differences (*p* = 0.044); however, Tukey's HSD test did not.

#### 3.1.2. Trial B

Water quality parameters for Trial B (Table 2) are presented as mean ± SD among treatments and include morning and evening dissolved oxygen (8.05 ± 0.52; 7.74 ± 0.49 mg L^−1^), temperature (24.71 ± 2.46°C), salinity (1.87 ± 0.39 ppt), pH (7.0 ± 0.6), total ammonia N (0.35 ± 0.33 mg L^−1^), and nitrite (0.44 ± 1.79 mg L^−1^). Results revealed no significant differences regardless of treatments across time and by week or time series analysis, in terms of water quality parameters evaluated: dissolved oxygen (mg L^−1^), salinity (ppt), pH, total ammonia N (mg L^−1^), and nitrite (mg L^−1^).

### 3.2. Growth Performance, Nutrient Retention, and Solids Management

#### 3.2.1. Trial A

Tilapia growth performance responses following feeding with two different probiotics (*B. velezensis* AP193 and *B. subtilis*) for 109 days are presented in Table 3. No differences in growth performance were detected across the dietary groups (*p* > 0.05). The ranges of growth parameters of tilapia fed with either probiotic or the commercial control diet were as follows: final biomass (26,456–27,767 g), weight gain (134.53%–149.17%), weight gain (165.96–179.89 g), FCR (1.27–1.44), net protein retention (28.45%–30.57%), and survival (89.17%–96.67%). Results from whole-body proximate analysis for Trial A (Table 3) revealed significant differences between moisture (67.27%–69.20%; *p* = 0.014), dry matter (32.47% −69.20%; *p* = 0.014), and protein DW (50.40% −55.50%; *p* = 0.003). However, no differences were detected in the fat and ash content (*p* > 0.05).

#### 3.2.2. Trial B

For Trial B, results revealed a similar trend following 90 days of culture (Table 4), showing no significant differences (*p* > 0.05) between treatments, and parameters ranges were as follows: final biomass (920–1130 g), weight gain (%) (45.95%–57.59%), weight gain (42.39–52.14 g), FCR (1.02–1.13), net protein retention (39.96%–51.18%), and survival (67.5%–97.5%). In Trial B, FCR (1.03–1.12; *p* = 0.005) showed significant differences between the commercial diet and the probiotic additions in the diet, regardless of the concentration. The data analyzed yielded no significant differences (*p* > 0.05) between treatments in moisture, dry matter, protein, fat, and ash. A one-way ANOVA demonstrated no significant differences (*p* > 0.05) for total solids (kg) ash (%), solids per unit of fish (kg), and solids per unit of feed input (kg) (Table 4).

### 3.3. Microbial Composition for Fecal Matter and Water Samples

Bacterial V3-V4 16S rRNA gene sequencing analyses were performed to characterize the microbial communities associated with biofloc water and fecal matter of fish. After read processing and chimera removal, the numbers of DNA sequences per sample are shown in Table 5.

#### 3.3.1. Trial A

**Figure 1:**
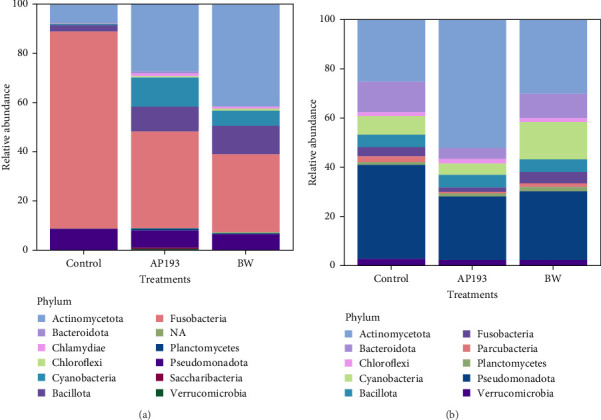
Relative abundance of bacterial phyla present in (A) fecal matter and (B) rearing system water for Nile tilapia (71.43 ± 4.44 g initial weight) fed a commercial feed (Control) alone, or top coated with BiOWiSH Feedbuilder Syn3 (BW) and AP193 over a 109-day production period.

The 16s rRNA gene sequencing analysis showed that the microbial community associated with fecal matter in fish was represented by a total of 12 phyla (Figure 1A) in Trial A, and biofloc water was represented by a total of 23 phyla. The 10 most common phyla are listed in Figure 1B. In Trial A, the bacterial phyla with the highest relative abundance were Fusobacteria and Actinomycetota in fecal samples and Actinomycetota and Pseudomonadota in water samples. These phyla were present in all samples across treatment groups. The phylum Bacillota, to which the genus *Bacillus*, which is the principal bacteria in the probiotics investigated, was also present in the study; nevertheless, these were found with a lower abundance compared to other phyla in commercial feed treated fish (2.63%), but within the fecal matter samples from the AP193-treated fish (10.08%) and BW-treated fish (11.58%) had a considerable abundance in the samples. However, after comparing the percentage of abundance by one-way ANOVA, there were no treatment differences (*p* = 0.210).

Bacterial alpha diversity, or within-sample diversity, of fecal matter and water, was assessed by observed species richness and Shannon diversity indexes. Shannon diversity varied moderately among treatment groups. In Trial A, fecal samples showed that AP193-treated fish contained the highest richness, while the Control diet-fed fish showed the lowest bacterial richness, though differences were not statistically significant (*p* = 0.717; Table 6). On the contrary, the most observed species in tank water was found when commercial feed was added, while water from AP193-treated tanks showed the lowest richness (*p* = 0.186; Table 6). Table 6 shows the average observed species richness and Shannon diversity indexes for bacterial communities in fecal matter and water samples in Trial A.

**Figure 2:**
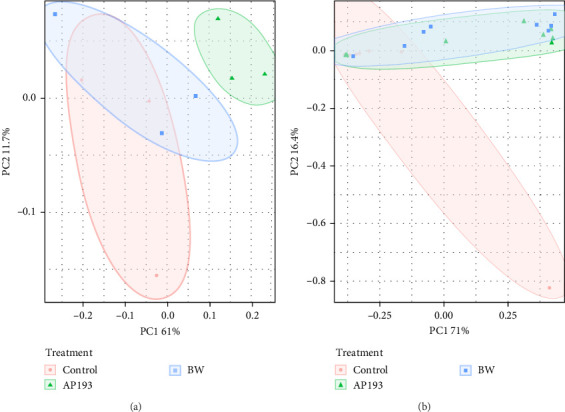
Principal coordinate analysis (PCoA) plots using weighted UniFrac beta diversity distances comparing (A) water samples (PERMANOVA *p* = 0.06, betadisper *p* = 0.691) and (B) fish fecal samples (PERMANOVA *p* = 0.015, betadisper *p* = 0.49) among treatments (Control, AP193, and BW).

The PERMANOVA, by treatment, conducted on weighted UniFrac dissimilarity matrices based on ASV level data, showed statistically significant differences (*p* = 0.015) in fecal matter samples in Trial A yielded. Subsequent pairwise-PERMANOVA post hoc analyses indicated that the fecal matter of fish fed with the Control treatment exhibited no statistically significant differences from those fed AP193 and BW (*p* = 0.067; Figure 2A).

When considering Bray–Curtis dissimilarity after agglomerating bacterial ASVs at the genus level, fecal samples in Trial A were found to be statistically different according to PERMANOVA (*p* = 0.008). The pairwise PERMANOVA post hoc results mirrored the observed trends found for Bray–Curtis at the ASV values. However, considering the phylogenetic similarity of ASV using either unweighted UniFrac or weighted UniFrac, no statistically significant differences were observed in fecal matter across the various treatments (*p* = 0.060).

In contrast, the beta diversity of water samples from Trial A did not reveal any statistically significant differences (*p* = 0.060) (Figure 2B) in terms of dispersion (betadisper) or group centroid (PERMANOVA). This lack of significance persisted when measuring with both weighted (ASV level) and unweighted UniFrac (ASV level), as well as Bray–Curtis dissimilarity metrics at both the ASV and genus level.

#### 3.3.2. Trial B

**Figure 3:**
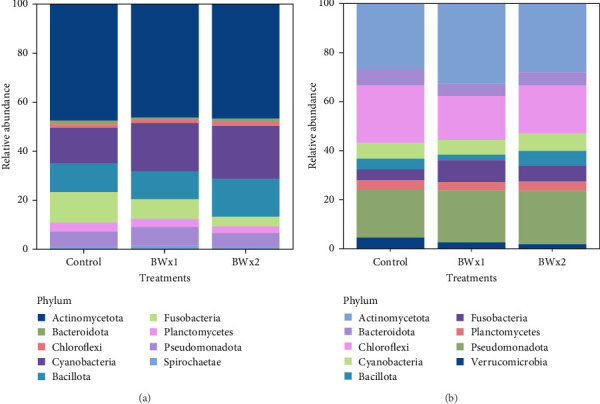
Relative abundance of bacterial phyla present in (A) fecal matter and (B) system water for Nile tilapia (5.34 ± 0.42 g initial weight) fed a commercial feed (Control) alone, or top coated with two concentrations of BiOWiSH Feedbuilder Syn3 over a 90-day production period.

**Figure 4:**
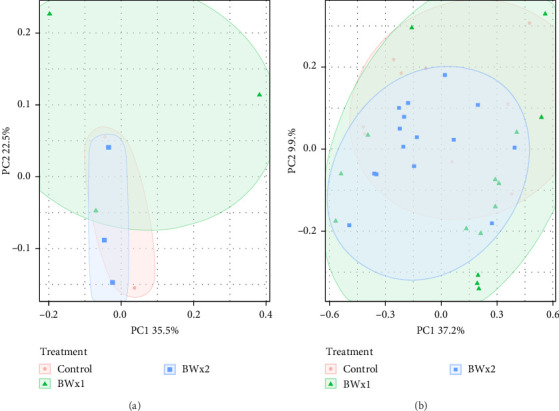
Principal coordinate analysis (PCoA) plots (based on weighted UniFrac distances) comparing (A) water samples (PERMANOVA *p* = 0.930, betadisper *p* = 0.274) and (B) fish fecal samples (PERMANOVA *p* = 0.056, betadisper *p* = 0.334) among treatments (Control, BWx1, BWx2).

In Trial B, 19 phyla were found in fecal samples and 27 phyla in biofloc water and the 10 most abundant phyla across all treatment groups are listed in Figures 3 and 4. The highest relative abundance in fecal matter across all treatment groups was Actinomycetota, and the second highest was Cyanobacteria. A similar trend was also observed in water samples, with Actinomycetota being the most abundant phylum across treatment. Pseudomonadota was observed as the second most abundant phyla in water samples from all treatments. Bacteria belonging to the genus *Bacillus*, the major bacterial strains found in BiOWiSH Feedbuilder Syn3, had a lower abundance in fecal samples. Tilapia fed the Control diet (5.67%) had a higher relative abundance compared to BWx1 (2.19%) but lower abundance than Bwx2-treated fish (7.38%). No statistically significant differences were found among the different BiOWiSH Feedbuilder Syn3 concentrations and the Control fish. However, after performing a pairwise *t*-test between the treatments, the relative abundance of bacteria from the genus *Bacillus* was increased in BWx2 compared to BWx1 (*p* = 0.004).

The same analysis approach was performed in fecal matter and water samples in Trial B. Comparisons of fecal matter bacterial richness showed that the BWx2 group contained the highest average number of observed species. At the same time, the lowest was found in the BWx1-fed tilapia. The alpha diversity of the BWx1 group was significantly lower than the other two groups (*p* = 0.020; Table 7). When the Shannon index was compared, no significant differences existed among the treatments (*p* = 0.180). When alpha diversity in terms of observed species and Shannon diversity index were compared in water samples for Trial B, no significant differences were observed among the treatments (*p* = 0.057) (Table 7).

In assessing various metrics for beta diversity in Trial B, there were no differences in terms of homogeneity of dispersion (*p* > 0.05) among treatments for both water samples (Figure 4A) and fecal samples (Figure 4B). However, upon conducting the PERMANOVA test, notable distinctions emerged. Specifically, significant differences were observed only in fecal samples in unweighted UniFrac values (*p* = 0.001), as well as Bray–Curtis at the ASV (*p* = 0.001) (Figure 4B) and genus level (*p* = 0.029).

## 4. Discussion

### 4.1. Growth Performance and Nutrient Retention

Biofloc production systems are a breakthrough technology that influences the environmentally conscious growth of aquaculture. This technology has been considered for tilapia culture due to the limitations of natural environments and the demands to increase productivity through high stocking densities [14]. Studies show that the growth of tilapia in BFT can potentially add to the nutritional intake of the fish when compared to more traditional culture methods [55, 56]. Thus, the combination of biofloc systems along with probiotics may be a viable method to improve fish culture and protect against disease outbreaks [57].

Different studies have demonstrated that probiotic applications increased growth performance, immune response, and improved water quality in both clearwater and biofloc aquaculture production systems [58–62]. In the current study, the use of *B. subtilis* and *B. velezensis* in both trials did not lead to improved growth performance of tilapia under the reported experimental conditions, but a reduction in FCR was observed with the probiotic addition in Trial B. This is consistent with a study by Nguyen et al. [63], which examined feed top coated with *B. velezensis* (AP193) and *B. subtilis* (BiOWiSH Feedbuilder Syn 3) in juvenile channel catfish (*Ictalurus punctalus*) reared in an indoor flow-through system with pond water. These authors found no significant difference in the growth performance of catfish after a 6-week feed trial and 8-week growth trial using three different concentrations of BiOWiSH Feedbuilder Syn3 top coated on commercial diets offered to channel catfish fingerlings. However, in Trial B of our study, an improvement in FCR was observed for both concentrations of BiOWiSH Feedbuilder Syn3, compared to a commercial diet. Abarike et al. [64] conducted a growth trial with Nile tilapia where fish were offered a combination of *B. subtilis* and *B. licheniformis* (1:1 ratio) with different dose concentrations for 4 weeks in a closed system. At the end of the trial, an improvement was observed in all probiotic treatments for FCR, final weight, and weight gain.

Al-Deriny et al. [65] conducted an aquarium study with Nile tilapia where *Spirulina plantensis* and *B. amyloliquefaciens* were offered to tilapia for 60 days and a combination of both probiotics. These authors found that weight gain was higher in fish fed *Spirulina* spp. and/or *Bacillus* spp. individually or in combination. Likewise, Thurlow et al. [66] carried out a 10-week feeding trial with channel catfish in a flow-through in-pond raceway system, fed with a commercial feed top coated with *B. velezensis*. In both aquaria and raceways systems, improvement in growth performance with the probiotic treatment was demonstrated in terms of weight gain. Our results do not align with what was reported in those studies, as no significant difference between probiotic supplementation and the commercial diet was noted. Nevertheless, the results observed in growth performance parameters, such as weight gain (%), FCR, and survival (%), showed promising trends.

Probiotics as dietary supplements can also influence the fish body composition. According to whole-body composition results of Trial A, significant differences were observed when tilapia were fed a commercial feed top coated with *B. velezensis* AP193 (Table 3). This confirms that the improved FCR is due to improved protein deposition. A 7-month growth trial with Nile tilapia was conducted by Opiyo et al. [67]; two different probiotics were offered to the Nile tilapia, including *Saccharomyces cerevisiae* and *B. subtilis*. The results demonstrated that varying levels of *B. subtilis* supplementation presented significant results in protein, moisture, and ash content. In Trial B, different BiOWiSH Feedbuilder Syn3 concentrations revealed no significant results in whole-body composition. Similarly, Reda and Selim [68] performed a 60-day aquarium growth trial with tilapia (*O. niloticus*), with 25% water exchange daily. The animals were offered a diet comprised of 39% protein and 10.9% fat and supplemented with *B. amyliquefaciens*. Probiotic supplementation also revealed no significant differences in moisture and ash content and presented lower protein retention (39.4%–43.6%).

### 4.2. Water Quality and Solids Management

In aquaculture systems, high stocking densities can be a problem for production due to the concentration of nitrogenous waste products in the system. However, in biofloc systems, bacteria in the floc help manage ammonia throughout the nitrification process, algal uptake, and bacterial assimilation. The *Bacillus* species contribute significantly to the reduction of nitrogenous and phosphorus components in the rearing water in addition to the maintenance of bacterial community structure equilibrium [43, 69, 70]. These interactions between bacteria and algae in the biofloc system are complex [10]. Using probiotics to help manage water quality, improve feed utilization, and decrease pathogenic microorganisms is a widespread but complex process [43, 68, 71, 72]. The current study showed no significant differences in either trial for most water quality parameters examined. It is worth noting that the total ammonia N concentration in Trial A peaked in all treatments from the 7th week to the 11th week, which occurred in parallel with an increase in feed allotment. El-Kady et al. [62] tested three different commercial probiotics. AquaStar (a mix of *Bacillus* spp., *Pediococcus* spp.), EM (a blend of *Rhodopseudomonas* spp., *Lactobacillus* spp., and *Saccharomyces* spp.), and MicroPan Complex (a union of *Bacillus* spp., and enzymes), administrated through the water body. The study was performed in concrete ponds with Nile tilapia fingerlings under natural conditions. After 60 days, water quality was improved due to decreased concentrations of total ammonia N in the system. The decrease of N compounds in the rearing water contributed to improved growth performance of the cultured fish.

Not only can probiotics influence water quality, but they also have the potential to degrade organic waste and reduce total solids buildup within the production system. Hence, we also examined discharged solids or effluent. A possible burden of the biofloc system design is the accumulation of solids, which can be harmful to cultured fish and lead to deterioration of water quality, which in turn decreases cultured species and production system performance [73–76]. For both trials in the current study, probiotics in the system showed no significant difference in total solids (g) compared to the commercial diet. However, there was a higher level of discharged solids in Trial B. Several factors influence waste produced in an intensive system production system such as biofloc. Due to excretion, microbe metabolism, and uneaten feed, the concentrations of phosphorus and N compounds can eventually present a problem when high levels are reached in the system [77]. Feed is the primary source of waste, and the nutrient content, quality, and quantity of feed will impact waste produced from dietary sources [78]. Hence, feeding protocols and the amount of feed inputs directly affect the concentration of solids or waste produced. The concentration of solids produced per kilogram of fish or feed was not significantly different between treatments in either trial. In our work, each trial was initiated with different-sized fish (Trial A: 71.43 ± 4.44 g; Trial B: 5.34 ± 0.42 g) and different seasons (Trial A was conducted in summer and Trial B in winter). We observed a trend between the amount of feed input and waste production, as the higher feed inputs resulted in higher nutrient loads, poorer water quality, and higher amounts of solids removed from the biofloc system. Effluent management helps regulate water quality and can be optimized to remain within a range most suited for the specific species cultured in the biofloc system. The goal is to maintain levels of suspended solids under the maximum level recommended [79].

### 4.3. Microbial Composition of Fish Fecal Matter and Water Samples

In aquatic environments, microorganisms play a crucial role in trophic networks [19]. They facilitate nutrient recirculation and interact with various organisms, making them an integral part of the ecosystem. To comprehend their specific niche and function, it is vital to identify and quantify community members. Next-generation DNA sequencing techniques have become an essential tool for studying noncultured microorganism communities [80]. This study describes the microbial community data in biofloc systems and how it changes when probiotics are added to biofloc systems. In Trial A, Fusobacteria and Actinomycetota were the most abundant taxa within the phyla detected in fish fecal matter (Figure 1A). These results were similar in the treatment and Control groups, indicating that they are typically in the tilapia GI tract. Apart from this, Bacillota were also the dominating phyla in BiOWiSH-treated fish. Fusobacteria, the most dominant phyla in the Control treatment group is a group of anaerobic bacteria commonly found in aquatic environments such as biofloc systems. In this study, Fusobacteria mainly consisted of *Cetobacterium* spp. This genus is an obligate gut associate and contributes to fish health by producing vitamin B12 [81].

Actinomycetota was the most dominant phyla in probiotic-treated groups and fecal matter of all treatment groups in Trial B (Table 7, Figure 1B). According to the results of Kathia et al. [82] and Abakari et al. [83], Actinomycetota is the most abundant bacterial species in tilapia raised in biofloc systems. One of the primary goals of this study was to examine how the addition of probiotics affected the microbial community in biofloc systems used for tilapia culture. Results revealed that the addition of probiotics did not significantly alter the existing microbial community of the water. This could be due to the addition of probiotics as dietary supplements instead of directly adding to the culture water. The relative abundance of *Bacillus* spp., which was a component of the two probiotics added, was low compared to other genera. It should be noted that when utilizing a biofloc culture system, the conditions for producing aquatic organisms are quite different from those of conventional systems. Biofloc systems promote the growth of a diverse range of heterotrophic bacteria through an external C source and aeration supply [84]. This can benefit the cultivated species and enable the growth of the most adaptable bacteria to this unique set of culture conditions [85, 86]. Some authors have also mentioned similar results upon the addition of probiotics containing *Bacillus* spp. and *Lactobacillus* spp. in biofloc systems which reared Pacific white shrimp (*Litopenaues vannamei*) and Nile tilapia [82, 87]. The fish and water microbiome may not have enough time to reach stable maturity due to the relatively short duration of the current study.

Results from Trial A indicate that bacterial diversity (Shannon index) in the fecal matter of fish treated with *B. velezensis* AP193 was 3.19, and BW was 3.36. These values were much higher compared to another previous study by Cardona et al. [88], which reared *Litopenaeus stylirostris* in a biofloc production system. The alpha diversity indices (Shannon and observed species) among the treatments in fecal matter and water samples showed no significant differences in Trial A. However, in Trial B, the number of observed species in fecal matter samples of BWx1 showed significantly lower values than the other two treatments. According to Borges et al. [89], when the probiotic concentration increases there can be an imbalance in the microbial composition in the fish GI tract. This could be the reason why BWx2 and commercial diet-fed fish had a similar number of observed species compared to BWx1. A significant difference was only seen in terms of observed species and not in the Shannon diversity index. Additional studies evaluating bacterial diversity composition on different probiotic dosages should be performed for more precise results. It is essential to mention that certain types of bacteria reside in the gut microbiota, and when exposed to water that is rich in carbohydrates, they can proliferate and grow significantly [90]. This growth occurs without the need for additional external sources of probiotics or other supplements and is instead fueled by the influx of C from carbohydrate-rich water. This could be why there were no significant differences in the growth performance of tilapia between the probiotic and nonprobiotic-treated groups [91].

Beta diversity measures the variation in species composition or community structure among different sites or samples within a larger ecosystem. It provides insights into how different environments or treatments influence the diversity and composition of species. Beta diversity complements alpha diversity (which measures diversity within a specific site). One commonly used metric for beta diversity is the Bray–Curtis dissimilarity index, which calculates the dissimilarity between two samples based on the abundance of different species. Other metrics include the Jaccard index, Sørensen index, and UniFrac distances [92]. In this experiment, the beta diversity of fish fecal matter and water samples were measured through the Bray–Curtis index and UniFrac distances, which are like Bray–Curtis but account for the phylogenetic relatedness of individual ASVs. In Trial A, the observed differences in fecal microbiota across diet treatments suggest that the type of diet influences the composition and structure of the tilapia gut microbiota. The significance of Bray–Curtis ASV and Bray–Curtis Genus metrics indicates that diet contributed to the observed variations in overall taxonomic composition. In Trial B, the absence of significant differences in multivariate dispersion suggests that the microbial communities have a similar level of multivariate variability within each treatment. The significant differences identified by PERMANOVA indicate that, despite similar variability, the overall composition of bacterial communities is distinct among treatments. In both trials, it is clear that the addition of probiotics can influence the fecal microbiota communities of Nile tilapia. Similar findings were reported by Siddik et al. [93] when juvenile barramundi (*Lates calcarifer*) was fed with a probiotic *S. cerevisiae* coupled with *Lactobacillus casei*. The authors also reported a significant impact on beta diversity in the fish gut microbiota. In another Nile tilapia study by Adeoye et al. [94], the PCoA (based on Bray–Curtis values) plot shows a spatial differentiation among the treatments (enzyme cocktail with probiotics containing *Bacillus* spp.).

The lack of significant differences in water samples in both feeding trials suggests that the treatment effects are more pronounced within the gut environment than in the surrounding water, which can be expected for a dietary-administered probiotic. The water microbiome of all treatments in Trial B has shifted compared to the time zero sampling point. It also implies that there are no systematic differences in water microbiota. The significant increases in beta diversity indicate the successful colonization or enrichment of the gut microbiota with beneficial microorganisms. This can include probiotic strains known for promoting health, improving digestion, or providing competitive exclusion against pathogenic bacteria. This information can guide the development of strategies to optimize probiotic use for improved aquaculture performance.

## 5. Conclusions

Tilapia production in biofloc production systems is an established and growing culture method used by commercial producers in many regions worldwide. Despite the widespread culture of tilapia using the biofloc production system, little information is available on the efficacy of dietary supplementation of probiotics on the growth performance of this species. Based on the observed findings of this and other studies, it can be quite challenging to predict the effects of supplementary probiotics on cultured aquatic species within a biofloc system. To further complicate matters, variability in production systems, husbandry techniques, and management can make comparisons between studies problematic. According to the findings of Trial B, the bacterial composition of fecal matter, as indicated by the Shannon index, improved when the recommended concentration of BiOWiSH Feedbuilder Syn3 was administered. However, there were no positive benefits to the growth performance of tilapia, water quality, or solids management. However, FCR was significantly better in fish treated with BWx1 and BWx2 when compared to the Control group. Thus, large-scale experimentation with various dosages under different production and husbandry conditions is required to establish appropriate protocols for using probiotics in biofloc systems to further optimize tilapia production.

## Figures and Tables

**Table 1:** Proximate composition of the 3 mm commercial diet in the feeding trial.

Parameters	g 100 g^−1^ (as is)
Crude protein^a^	41.01
Moisture	5.81
Crude fat	11.64
Fat (acid hydrolysis)	13.75
Crude fiber	1.79
Ash	6.61
Phosphorus	1.17

*Note:* Analysis conducted by the University of Missouri Agricultural Experimental Station Chemical Laboratories (Columbia, MO, USA) (results are expressed on g/100g of feed as is, unless otherwise indicated). Results are expressed on an “as is” basis unless otherwise indicated.

^a^Crude protein = %*N* × 6.25; estimates provided. § nonproteinogenic amino acids.

**Table 2:** Water quality parameters throughout the two Nile tilapia feeding trials.

Trial A	Control	BW	AP193	PSE	*p*-Value
Morning DO (mg L^−1^)	7.47 ± 0.62	7.41 ± 0.61	7.44 ± 0.67	0.27	0.543
Evening DO (mg L^−1^)	7.23 ± 1.4	7.16 ± 1.47	7.21 ± 1.38	0.40	0.832
Temperature (°C)	26.64 ± 1.82^b^	26.90 ± 2.14^a^	26.56 ± 1.76^b^	0.42	0.040
pH	6.84 ± 0.28	6.89 ± 0.62	6.95 ± 0.92	0.27	0.795
Salinity (mg L^−1^)	0.96 ± 0.44	1.0 ± 0.46	0.94 ± 0.42	0.20	0.244
TAN (mg L^−1^)	8.56 ± 6.75^a^	8.31 ± 5.26^a^	4.73 ± 11.39^a^	2.77	0.044
UA (mg L^−1^)	3.8 ± 4.73^a^	3.53 ± 4.36^a^	2.14 ± 3.2^b^	1.39	0.044
Nitrite (mg L^−1^)	0.58 ± 0.42	0.51 ± 0.67	0.63 ± 0.38	0.17	0.594

**Trial B**	**Control**	**BWx1**	**BWx2**	**PSE**	***p*-Value**

Morning DO (mg L^−1^)	8.07 ± 0.51	8.04 ± 0.53	8.04 ± 0.50	20.04	0.437
Evening DO (mg L^−1^)	7.77 ± 0.47	7.73 ± 0.49	7.72 ± 0.47	0.29	0.552
Temperature (°C)	24.8 ± 2.31	24.73 ± 2.37	24.73 ± 2.46	1.46	0.948
pH	6.87 ± 0.57	7.00 ± 0.51	7.03 ± 0.45	0.31	0.161
Salinity (mg L^−1^)	1.84 ± 0.22	1.81 ± 0.24	1.89 ± 0.48	0.21	0.295
TAN (mg L^−1^)	0.28 ± 0.18	0.36 ± 0.29	0.27 ± 0.23	0.15	0.085
UA (mg L^−1^)	0.14 ± 0.09	0.18 ± 0.15	0.14 ± 0.11	0.15	0.085
Nitrite (mg L^−1^)	0.23 ± 0.22	0.73 ± 2.99	0.29 ± 0.26	1.13	0.281

*Note:* Different letters denote statistical differences among treatment groups. Water quality parameters throughout 109 days of rearing tilapia (*Oreochromis niloticus*; 71.43 ± 4.44 g) in a biofloc system and offered a commercial feed (control) top coated with either AP193 or BiOWiSH Feedbuilder Syn3 (Trial A). Water quality parameters throughout 90 days of rearing juvenile tilapia (5.34 ± 0.42 g) in a biofloc system, and fed with two different concentrations of BiOWiSH Feedbuilder Syn3, compared to a commercial, control diet (Trial B). Values are presented as the mean ± standard deviation. Different letters denote statistical differences among treatment groups.

Abbreviations: PSE, pooled standard error; TAN, total ammonia nitrogen, UA, unionized ammonia.

**Table 3:** Response of tilapia (71.43 ± 4.44 g) reared over a 109-day culture period in individual biofloc type systems and offered a commercial feed or one top coated with BiOWiSH Feedbuilder Syn3 (BW), or AP193.

Parameter	Control	BW	AP193	PSE	*p*-Value
*Growth performance*					
Final biomass (kg)	26.46	27.77	26.46	0.668	0.736
Final mean weight (g)	252.9	239.98	236.75	5.360	0.093
Weight gain (g)	179.89	171.11	165.96	6.130	0.278
Weight gain (%)	148.70	149.17	134.53	0.110	0.760
FCR	1.38	1.27	1.44	0.110	0.586
ANPR (%)	30.57	28.45	28.95	0.560	0.658
Survival (%)	89.17	96.67	96.67	4.000	0.373
*Discharged solids*					
Total solids (kg)	217.3	141.79	173.97	18.03	0.299
Solids per unit of fish (kg)	8.14	6.52	5.31	0.68	0.306
Solids per unit of feed input (kg)	7.99	4.97	6.96	0.72	0.294
Ash (%)	82.82	82.94	81.09	0.330	0.101
*Whole-body proximate composition*					
Moisture (%)	67.27^b^	67.53^b^	69.20^a^	0.200	0.014
Dry matter (%)	32.73^a^	32.47^a^	30.80^b^	0.200	0.014
Protein DW (%)	50.40^b^	50.40^b^	55.50^a^	0.390	0.003
Fat DW (%)	31.03	31.17	32	1.210	0.941
Ash DW (%)	16.33	16.73	12.60	0.670	0.086

*Note*: Different letters denote statistical differences among treatment groups.

Abbreviations: ANPR, apparent net protein retention; DW, dry weight; PSE, pooled standard error.

**Table 4:** Response of juvenile tilapia (5.34 ± 0.42 g) reared over 90 days of culture period in individual biofloc type systems and offered a commercial diet (control) with two inclusion levels of BiOWiSH Feedbuilder Syn3 (BWx1 and BWx2), top coated to the diets.

Parameter	Control	BWx1	BWx2	PSE	*p*-Value
*Growth performance*					
Final biomass (kg)	7.8	9.16	9.03	0.483	0.291
Final mean weight (g)	53	53	50	2.90	0.796
Weight gain (g)	48	47	45	2.94	0.767
Weight gain (%)	968	887	884	55.27	0.393
FCR	1.12^a^	1.05^b^	1.03^b^	0.01	0.005
ANPR (%)	25.95	23.63	23.26	1.12	0.325
Survival (%)	76	87	90	4.33	0.167
*Discharged solids*					
Total solids (kg)	2752	2351	1317	729.38	0.426
Solids per unit of fish (kg)	370.30	271.00	160.3	91.60	0.371
Solids per unit of feed input (kg)	301.40	274.90	159.3	91.43	0.540
Ash (%)	31.17	28.77	28.51	1.97	0.268
*Whole-body proximate composition*					
Moisture (%)	72.65	71	72.27	0.1269	0.220
Dry matter (%)	27.35	29	27.73	0.1269	0.220
Protein DW (%)	55.5	52	54.57	0.0712	0.220
Fat DW (%)	26.9	30.23	29.43	0.1134	0.139
Ash DW (%)	12.30	12.03	13.27	0.5373	0.430

*Note*: Different letters denote statistical differences among treatment groups.

Abbreviations: ANPR, apparent net protein retention; DW, dry weight; PSE, pooled standard error.

**Table 5:** DNA sequences per sample following chimera removal in Trials A and B.

Experiment	Fecal samples	Water samples
Trial A	39,241 ± 11,081	39,103 ± 5691
Trial B	192,640 ± 25,387	188,654 ± 19,555

**Table 6:** Average observed number of species and Shannon diversity indices of bacterial communities in fecal matter and water for tilapia (*O. niloticus*; 71.43 ± 4.44 g) over a 109-day production period.

Treatment	Shannon	Observed number of species	Global ANOVA *p*-value
*Fecal matter*			*p* = 0.717
AP193	3.19 ± 1.68	70.53 ± 47.97	
BW	3.36 ± 0.64	58.39 ± 13.43	
Control	1.82 ± 0.59	34.19 ± 30.68	
*Water samples*			*p* = 0.186
AP193	4.70 ± 0.16	141.72 ± 21.16	
BW	5.12 ± 0.23	173.87 ± 8.71	
Control	5.28 ± 0.64	216.93 ± 72.09	

*Note:* Tilapia were fed a commercial feed top coated with BiOWiSH Feedbuilder Syn3 (BW) and AP193, compared to the commercial diet.

**Table 7:** Average bacterial richness (observed species) and Shannon diversity index of bacterial communities identified in fecal matter and water for tilapia (*O. niloticus*; 5.34 ± 0.42 g) over a 90-day production period.

Treatment	Shannon	Observed number of species	Global ANOVA *p*-value
*Fecal matter*			
BWx1	4.28 ± 0.11	180.11 ± 4.66	*p* = 0.020
BWx2	4.58 ± 0.26	228.63 ± 17.08	
Control	4.66 ± 0.21	227.73 ± 22.20	
*Water samples*			
BWx1	5.66 ± 0.39	362.26 ± 30.36	*p* = 0.057
BWx2	5.64 ± 0.09	404.87 ± 20.95	
Control	5.77 ± 0.62	429.25 ± 2.91	

*Note:* Tilapia were fed a commercial feed top coated with two concentrations of BiOWiSH Feedbuilder Syn3 (BWx1 and BWx2) added to a commercial diet (Control).

## Data Availability

The data that support the findings of this study are available from the corresponding author upon reasonable request.
